# Molecular Dynamics Study of the Influence of Nano SiO_2_ on the Thermodynamic Properties of PMIA Composites

**DOI:** 10.3390/polym14153134

**Published:** 2022-08-01

**Authors:** Bowen Liu, Fangcheng Lv, Xiaozhou Fan, Yuxuan Li, Bowen Jiang

**Affiliations:** Hebei Provincial Key Laboratory of Power Transmission Equipment Security Defence, North China Electric Power University, Baoding 071003, China; lbw@ncepu.edu.cn (B.L.); lfc@ncepu.edu.cn (F.L.); lyxncepu@ncepu.edu.cn (Y.L.); jbowen@ncepu.edu.cn (B.J.)

**Keywords:** PMIA, thermal conductivity, thermodynamic properties, molecular dynamics simulation, radial distribution function

## Abstract

The poly-m-phenyleneisophthalamide (PMIA) is widely used in the electrical field due to its numerous favorable characteristics, but its poor thermal conductivity limits its application. In this study, PMIA was modified with nano-silica (SiO_2_) to improve its thermal and mechanical properties. Using iso-phthalic acid and m-phenylenediamine as monomers, the changes in the thermodynamic properties and microstructure parameters of SiO_2_-modified PMIA were analyzed using molecular dynamics before and after modification in the temperature range of 250~450 K. It was found that adding SiO_2_ improves the Young’s modulus and Shear modulus of PMIA, and the mechanical properties of PMIA, and SiO_2_/PMIA composites deteriorate with increasing temperature, but the mechanical properties of SiO_2_/PMIA composites are always better than those of pure PMIA in the temperature range of electrical equipment. Meanwhile, after doping SiO_2_ with the radius of 8 Å, the glass transition temperature of PMIA increases by 27.11 K, and its thermal conductivity increases from 0.249 W m^−1^ K^−1^ to 0.396 W m^−1^ K^−1^. When SiO_2_ is added to PMIA, the thermal expansion coefficient of PMIA will decrease in both glass and rubber states, and its thermal stability will improve. In terms of microstructure parameters, the free volume distribution of the SiO_2_/PMIA model is less easily dispersed than that of the PMIA model, indicating that the addition of SiO_2_ can improve the related properties of PMIA by hindering the movement of molecular chains.

## 1. Introduction

As the most critical and expensive equipment in the power system, the health of the transformer is an important guarantee of the economic operation, and the safety and stability of the power grid [1]. The insulating materials play a decisive role in the safety of equipment, and the man-made fibers have obvious advantages in thermal stability, electrical properties, and mechanical properties compared with natural fiber products [2], which can greatly improve the safety of electrical appliances.

Aramid paper series products play an important role in insulation materials, including meta-aramid paper, aramid-mica paper, and aramid soft composite materials [3]. Among these special insulation papers, PMIA insulation paper products are widely used. PMIA is a linear macromolecular fiber composed of aromatic groups linked by amide bonds. It has the advantages of solid flame retardancy, high elastic modulus, strong electrical insulation, high-temperature resistance, and high strength [4,5], making it more suitable for transformers and other electrical equipment. However, the thermal conductivity of pure PMIA is relatively poor, mostly about 0.24 W m^−1^ K^−1^ [6], making it hard to dissipate heat in bushings and other parts of transformers in time, thus causing adverse effects on insulation and hindering its application in the power system. Therefore, PMIA needs to be further processed to be better applied to electrical equipment.

At present, there are two main approaches to improving the thermal conductivity of polymers. One is to improve the intrinsic thermal conductivity of polymers from the polymer matrix. The second is to prepare polymer composites by adding high thermal conductivity fillers, such as Al, Al_2_O_3_, BN, carbon nanotubes, graphene, etc., to the polymer matrix [7,8]. Among them, nano-SiO_2_ filler is cheap and has excellent thermal and mechanical properties. When it is added into polymer, the composites formed not only have high insulation strength, but also have high thermal conductivity and heat resistance of inorganic filler [9]. Lihua Wang and other scholars doped silica nanoparticles into insulating paper cellulose to improve its mechanical properties and glassy transition temperature [10]. Zhikun Wang and others modified nano-silica doped epoxy resin with silane coupling agent [11]. It was found that the glass transition temperature of epoxy resin increased by 21 K after doping nano-silica particles. The above research fully shows that SiO_2_ can improve the thermal conductivity and mechanical properties of polymer materials, but the mechanism by which SiO_2_ improves the thermodynamic properties of PMIA needs further study.

Molecular dynamics (MD) simulations have been used to study the behavior of molecules at the atomic/molecular level, which can be very convenient to study the changes in the microstructural parameters of materials, establish the relationship between micro and macroscopic, and provide a more reasonable explanation of the interaction mechanism between fillers and substrates [12,13].

In this paper, the models of PMIA and SiO_2_-doped aramid composite (SiO_2_/PMIA) were established using molecular dynamics simulation. The changes in thermal conductivity, glass transition temperature, thermal expansion coefficient, and the mechanical properties of PMIA doped with SiO_2_ were studied and analyzed, and the macroscopic properties were explained by microscopic parameters such as free volume and radial distribution function. At the same time, the influence of temperature on the properties of meta-aramid and SiO_2_/PMIA composites in the temperature range of power equipment was studied, which laid a foundation for the subsequent preparation of high thermal conductivity aramid materials suitable for the power system.

## 2. Modeling and Dynamic Optimization

Models were built in Materials Studio (MS) software [14]. All simulations were carried out in the Focite and Amorphous Cell modules included in the (MS) software.

### 2.1. Establishment of PMIA Monomer Model

**Figure 1:**
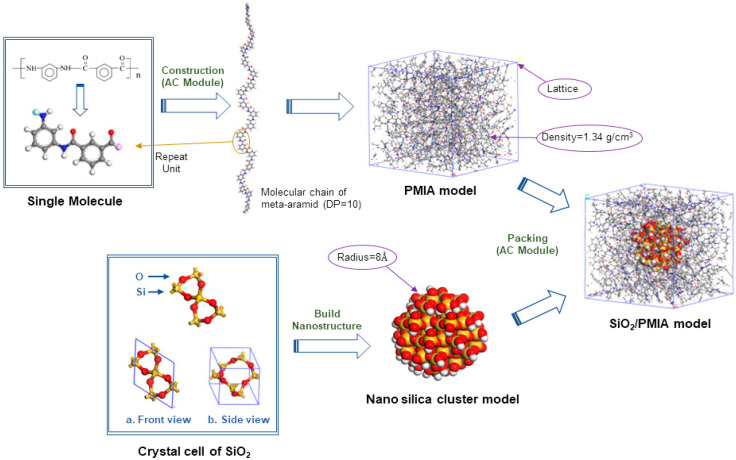
Establishment of the pure PMIA and SiO_2_/PMIA models.

PMIA is a polymer with a molecular weight of 60,000–90,000 formed by spatial intricate polymerization of Iso-phthalic Acid and m-Phenylenediamine monomers [15]. The molecular model of PMIA monomer is shown in Figure 1. In order to make the established model correspond to a real situation, periodic boundary conditions were used in this simulation. Although PMIA is artificially synthesized, its structure includes crystalline region (shaped region) and amorphous region (unshaped region) just like cellulose in natural state. Results show that the crystallinity of PMIA is very low, and the amorphous region accounts for 75~80% [16]. An aramid fiber chain may pass through several shaped areas and amorphous areas. Therefore, the length of the fiber chain in the amorphous region may be very short. At the same time, the research shows that there is no obvious difference in molecular conformation or physical and chemical properties among the amorphous fiber models composed of different fiber chain lengths [16], so the molecular polymerization degree of the constructed PMIA is 10 [17,18].

### 2.2. Establishment of Nano-SiO_2_ Model

Nano-silica is characterized by high surface activity, poor stability, unsaturated residual bonds, and highly polar hydroxyl groups [19,20]. When building the SiO_2_ nano-cluster model by Build module in the MS software, the surface of the SiO_2_ molecule was oxidized, that is, the broken bond on the Si atom on the surface is combined with -OH, and the broken bond on the O atom on the surface is combined with -H atom. The radius of SiO_2_ nanoclusters is set to 8 Å. The initial configuration of SiO_2_ crystal is not stable, so it is necessary to perform the geometric optimization in the Forcite module of the MS software to obtain its low-energy morphology.

### 2.3. Establishment of PMIA/SiO_2_ Composite Model

PMIA crystal cell and SiO_2_-doped PMIA/SiO_2_ composite crystal cell were established by the Amorphous Cell module of the MS software, as shown in Figure 1. The real density of aramid fiber is close to 1.34 g/cm^3^, but to avoid the penetration and connection between aramid molecular chains during the establishment of single cells, a lower initial density should be selected so that the molecular chains have enough space to twist [21]. After several calculations, when the initial density of the model is set to 0.1 g/cm^3^, it takes too long to perform simulation under NPT ensemble several times to increase the density. Set the initial density to 0.6 g/cm^3^, set the pressure to 0.15 GPa, and 100 ps simulation under NPT ensemble is carried out to make the cell density close to the experimental value.

### 2.4. Geometric Optimization and Annealing Processes

The Focite module is selected to optimize the geometry of the model, to obtain its low-energy configuration. Then, to make the hole distribution of the crystal cell closer to the real material and overcome the potential well between the local minimums of the system, in the Focite module, the Anneal task is selected to carry out 25 cyclic annealing treatments from 800 K to 300 K, and then to 800 K. After the above treatment, the local unreasonable structure in the amorphous model is eliminated, making the molecular unit cell more balanced and stable, which provides a reasonable geometric conformation for the next simulation calculation.

The COMPASS force field is used in all modeling and geometric optimization processes. This force field has proved to be the first molecular force field that unifies organic and inorganic molecules into one force field [22]. The Andersen and Berendsen methods are used to control temperature and pressure, respectively. The Van der Waals and electrostatic interactions are controlled by Atom-Based and Ewald methods, respectively; the van der Waals force cutoff value is set to 10 Å. The Maxwell distribution is used to randomly assign the initial velocity of atoms. Boundaries are controlled using periodic boundaries.

## 3. Calculation of Thermodynamic Performance

### 3.1. Calculation of Thermal Conductivity

**Figure 2:**
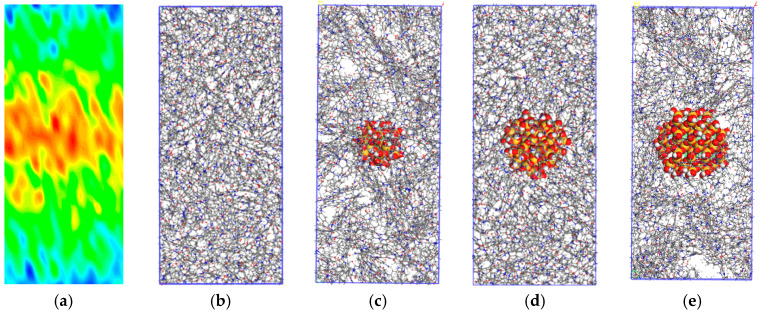
Establishment of thermal conductivity model and temperature distribution (**a**) The temperature distribution of the model; (**b**) PMIA model for heat conduction calculation; (**c**) SiO_2_/PMIA model for heat conduction calculation (4 Å); (**d**) SiO_2_/PMIA model for heat conduction calculation (8 Å); (**e**) SiO_2_/PMIA model for heat conduction calculation (12 Å).

The equilibrium model is simulated by the RNEMD (Reverse Non-Equilibrium Molecular Dynamics) [23] method with LAMMPS (Large-scale Atomic/Molecular Massively Parallel Simulator) software [24], and periodic boundaries are applied in x, y, and z directions. The RNEMD method uniformly divides the model into several layers (20 layers are divided in this paper) along a certain direction (z direction is selected in this paper); the layers on both sides are called “cold layer” and the middle layer is called “hot layer”. The Langevin temperature control method is used to keep the two regions at preset temperatures (330 K in hot domain and 270 K in cold domain), as shown in Figure 2. By exchanging the kinetic energy between the coldest particles in the hot layer and the hottest particles in the cold layer, the energy exchange is realized. By exchanging energy many times and taking the average value, a stable temperature gradient is finally formed in the system [25]. The calculation of thermal conductivity is based on the Fourier heat transfer law:

(1) $$\lambda =-\frac{J}{dT/dz}$$

where *J* is the energy flux in the z direction, *dT*/*dz* is the temperature gradient, and the minus sign indicates that the direction of the energy flux is opposite to the gradient.

Then, a 500 ps RNEMD simulation under NVT ensemble is carried out. In this paper, the time step of all simulation processes is 0.1 fs. The first 250 ps is used for system equilibrium, and the last 250 ps is used for result analysis. The temperature in this time range is counted for 10 times (50 ps/time), and the mathematical average of the 10 results is calculated. The temperature drop Δ*T* at the interface is obtained by fitting the linear part of the temperature in the polymer region. Finally, the interfacial thermal conductivity is calculated by Formula (2):

(2) $$J_{Q}=-G_{k}\cdot \Delta T$$

ΔT represents the interface temperature drop after passing through the interface, and the $J_{Q}=\left(\Delta E_{1}+\Delta E_{2}\right)/2A\Delta t$ represents heat flow, $\Delta E_{1}$ and $\Delta E_{2}$ is the energy of the hot domain and the energy of the cold domain respectively; *A* represents cross-sectional area at the interface; Δt represents simulation time.

To divide the layers conveniently, the model is expanded about three times along the z direction in a molecular dynamics calculation, so it is necessary to establish the PMIA and SiO_2_/PMIA models for calculating thermal conductivity separately, according to the previous model construction method. When a stable temperature gradient is formed in the system, the model temperature changes along the z direction as shown in Figure 2, with the highest temperature at both ends of the model, and gradually decreasing along the z direction to the middle.

**Figure 3:**
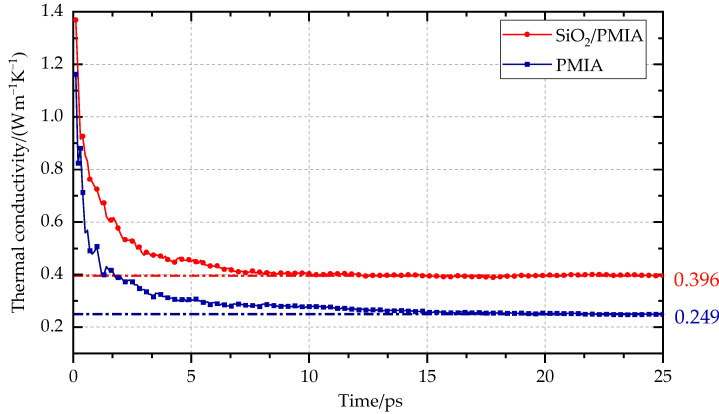
Thermal conductivity simulation results.

The results in Figure 3 show that the thermal conductivity of PMIA increases to a certain extent after SiO_2_ modification. The thermal conductivity of pure PMIA is 0.249 W m^−1^ K^−1^, and the experimental value of thermal conductivity of PMIA is about 0.24 W m^−1^ K^−1^ from the literature [6]. The comparison shows that the simulation result is close to the experimental value, and the relative error is 3.75%. This indicates that the molecular dynamics simulation results are reliable within the allowed range.

It can be seen from Table 1 that with the increase of the nano-SiO_2_ radius, the thermal conductivity first increases and then decreases, reaching the maximum value when the radius is 8 Å. At this time, the thermal conductivity of SiO_2_/PMIA is 0.396 W m^−1^ K^−1^, which is 59.04% higher than that of pure PMIA.

### 3.2. Coefficient of Thermal Expansion

The object will shrink and expand with the change of temperature. The coefficient of thermal expansion (CTE) refers to the ratio of the length change in one direction of a solid substance to its length at room temperature when the temperature changes by 1 K [26]. For most objects, CTE is positive, that is, its length and volume will increase with the increase of temperature. CTE is an important index to measure the thermal stability of materials [27], and the formula for obtaining CTE is as follows:

(3) $$\alpha =\frac{1}{V}{\left(\frac{\partial V}{\partial T}\right)}_{p}$$

(4) $$\beta =\frac{1}{3}\alpha$$

**Figure 4:**
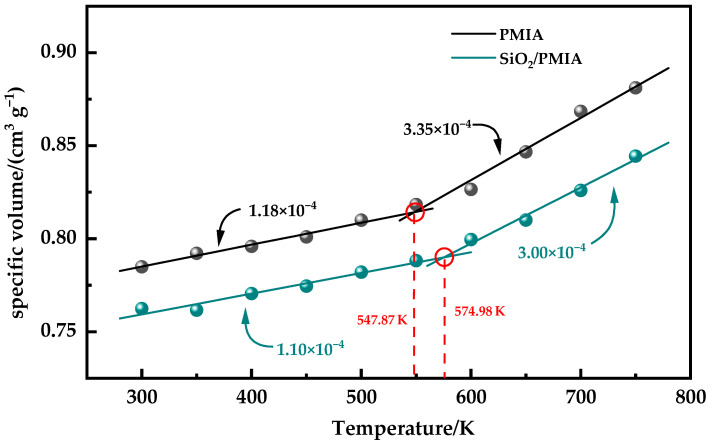
Coefficient of the thermal expansion of the model.

The glass transition temperature (*Tg*) is the boundary point between the glassy state and the rubber state of materials, which can be used as a characteristic index to characterize polymers. The density, volume, and other related parameters change greatly before and after the glass transition temperature. The volume and temperature of the system before and after the glass transition temperature are fitted to obtain the corresponding temperature volume curve, as shown in Figure 4. It can be seen from Figure 4 that there is an obvious inflection point in the volume temperature curve. Taking this inflection point as the dividing point, the volume temperature data before and after the inflection point are linearly fitted. The intersection of the two fitting curves is the glass transition temperature. The slope of the fitting curve before and after the glass transition temperature of PMIA and SiO_2_/PMIA systems is obtained from the fitting curve, and then the thermal expansion coefficients of the glass state (below *Tg*) and the rubber state (above *Tg*) of each system are calculated through Formulas (3) and (4).

It can be seen from Figure 4 that the CTE of the system in the glass state is smaller than that in the rubber state, because the free volume remains unchanged in the glass state and the mobility of molecular segments is weak, while the temperature in the rubber state is relatively high, the intermolecular force is weakened, and the mobility of molecules is enhanced, so the thermal stability of aramid materials in the glass state is better than that in the rubber state. After the addition of SiO_2_, the CTE of PMIA will decrease in both glass and rubber states, and the thermal stability of the composite will be improved.

### 3.3. Mechanical Parameters

The main function of aramid insulation paper in electrical equipment is to be the electrical insulation of a copper conductor. If the performance of aramid insulation paper wound around the copper conductor is poor, the insulation paper will be easily damaged when the conductor coil is subjected to mechanical stress, and this kind of damage is irreversible, which will greatly reduce the performance of the insulation paper. Therefore, the improvement of mechanical properties of the insulation paper can not only indirectly improve the performance of the insulation paper, but also prolong the service life of electrical equipment in the power system.

Hooke’s law can be used to express the strain in solid materials $\varepsilon_{j}$ and stress $\sigma_{i}$ [28]:

(5) $$\sigma_{i}=C_{ij}\varepsilon_{j}$$

where $C_{ij}$ is 6 × 6 elastic stiffness coefficient matrix. Theoretically, all mechanical properties can be derived by $C_{ij}$ .

It is found that the elastic strain of most materials is symmetrical, that is, $C_{ij}=C_{ji}$ in the matrix. For isotropic materials within the error range, the elastic stiffness matrix can be simplified as:

(6) $$C=\left[\begin{array}{cccccc} \lambda +\mu & \lambda & \lambda & 0 & 0 & 0\\ \lambda & \lambda +\mu & \lambda & 0 & 0 & 0\\ \lambda & \lambda & \lambda +\mu & 0 & 0 & 0\\ 0 & 0 & 0 & \mu & 0 & 0\\ 0 & 0 & 0 & 0 & \mu & 0\\ 0 & 0 & 0 & 0 & 0 & \mu \end{array}\right]$$

(7) $$E=\mu \frac{3\lambda +2\mu }{\lambda +\mu }$$

(8) G=μ

Lamé constant λ and μ are all constants, which can be obtained by molecular dynamics calculation. The elastic modulus *E* and Shear modulus *G* of material mechanical parameters can be obtained from Equations (7) and (8) respectively.

**Figure 5:**
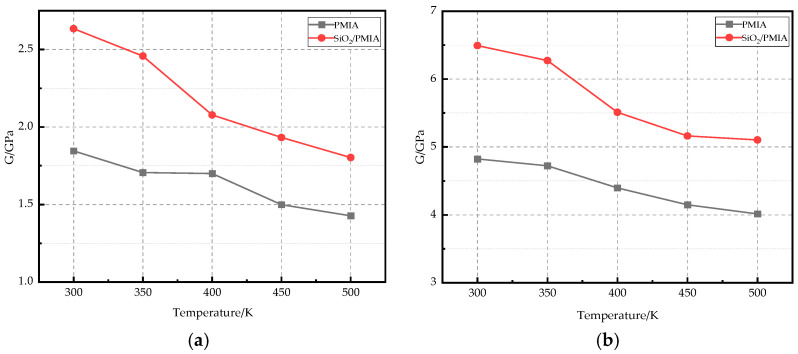
Mechanical properties at different temperatures. (**a**) Young’s modulus, (**b**) Shear modulus.

The PMIA and SiO_2_/PMIA models at 250 K, 300 K, 350 K, 400 K, and 450 K after cooling simulation were selected for geometric optimization again, and the optimized unit cells were selected to eliminate the internal stress of the system as much as possible. After geometrical optimization, the NPT dynamics of the model is calculated for 50 ps again; one model is output every 1 ps, until finally 50 models are output. The five models with the lowest potential energy are selected to calculate their mechanical properties. The constant strain method is selected for calculation, and the maximum strain is 0.003. Finally, the sum of Lamé constants of the PMIA and SiO_2_/PMIA models at different temperatures is obtained, and then five groups of mechanical parameters of corresponding models are obtained from equations, and their average values are taken as mechanical parameters at each temperature. Figure 5 shows the variation of the Young’s modulus and Shear modulus of pure PMIA and SiO_2_/PMIA material with temperature.

It can be seen from Figure 5 that the Young’s modulus and Shear modulus of PMIA increase after adding SiO_2_, which shows that adding SiO_2_ can enhance the rigidity of aramid material and make it less prone to deformation. As the temperature increases, the Young’s modulus and Shear modulus of pure PMIA and SiO_2_/PMIA composites decreased. The Young’s modulus of pure PMIA at 450 K decreases by 16.8% compared with that of 250 K, and the Shear modulus decreased by 16.8%. However, the Young’s modulus and Shear modulus of SiO_2_/PMIA composites decreased by 20.9% and 22.2%, respectively. The stiffness of PMIA and SiO_2_/PMIA composites decreased with the increase of temperature, and the mechanical properties of SiO_2_/PMIA composites were more affected by the increase of temperature. However, the mechanical properties of PMIAs with added SiO_2_ were always better than those of pure PMIAs in the temperature range of electrical equipment.

## 4. Calculation of Structural Parameters

### 4.1. Proportion of Free Volume

According to the free volume theory, the holes between molecules in materials are called free volumes, and the space occupied by molecular chains is called occupied volumes [29]. Free volumes provide active space for the movement of molecular chains. The fractional free volume (FFV) and free volume size of amorphous polymer materials affect the properties of polymer materials, such as glass transition temperature.

**Figure 6:**
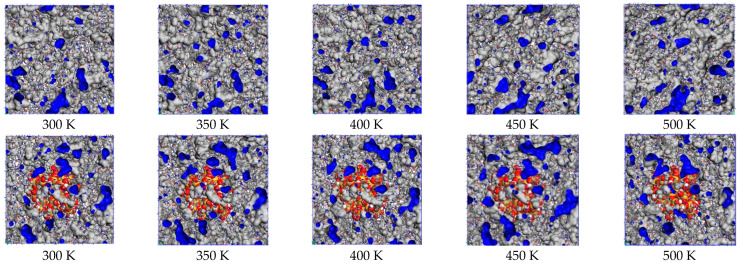
Free volume distribution of the model at different temperatures.

The free volumes and occupied volumes of the PMIA and SiO_2_/PMIA models at different temperatures are shown in the table. Figure 6 shows the free volumes of the PMIA and SiO_2_/PMIA models at different temperatures (blue part). It can be seen from Figure 6 that with the increase of temperature, the area occupied by free volumes gradually increases, which is consistent with the calculation result of free volume ratio in Table 2. In the pure meta-aramid model, with the increase of temperature, the free volume in the model begins to disperse gradually, which is because the increase of temperature leads to the acceleration of molecular chain movement, the increase of intermolecular distance, and the greater dispersion of molecular chains. After adding SiO_2_, the free volume distribution in the SiO_2_/PMIA model is not particularly dispersed with the increase of temperature, which further shows that adding SiO_2_ will hinder the movement of the aramid molecular chain.

When aramid is doped with SiO_2_, it increases the density of the system, which makes the material more tightly bound internally, reduces the gaps between the aramid molecular chains, and increases the resistance to movement, thus weakening the chain motion of the aramid molecular chains.

**Figure 7:**
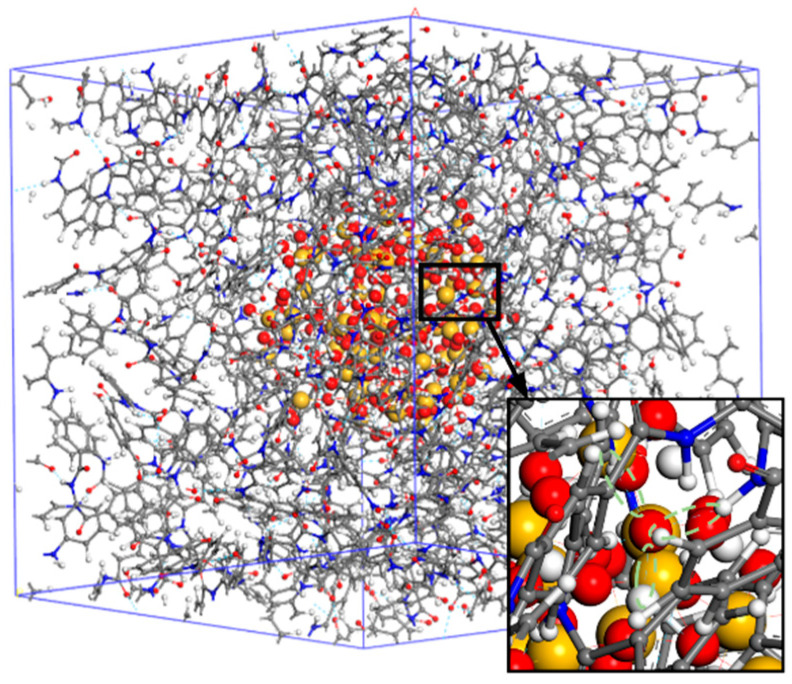
Local enlargement of hydrogen bonding in the SiO_2_/PMIA model.

The presence of hydrogen bonds is one of the reasons for the tight junctions of aramid fiber molecules. The addition of nano-silica, which contains a large number of hydroxyl oxygen atoms and molecules, easily combines with the -NH group and oxygen atoms in the aramid fiber, creating new hydrogen bonds, as shown in Figure 7. Due to the formation of a new hydrogen bonding network, the whole system is more compact. Theoretically, this reduces the motility of the aramid fibers and thus reduces the FFV of the aramid fibers.

### 4.2. Radial Distribution Function

**Figure 8:**
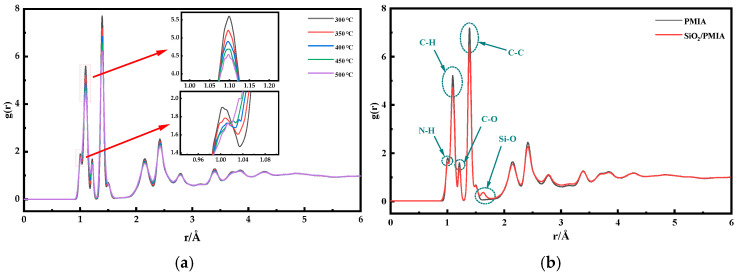
RDF of total atoms of the system (**a**) PMIA system in different temperature; (**b**) PMIA and SiO_2_/PMIA systems.

**Figure 9:**
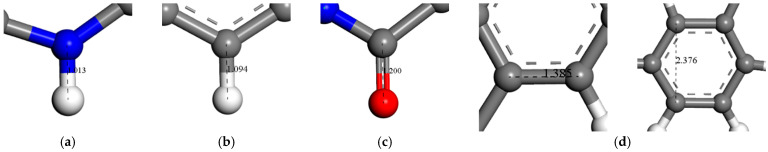
Schematic diagram of local structure (**a**) Covalent bond between N and H; (**b**) Covalent bond between C and H; (**c**) Covalent bond between C and O; (**d**) Distance between C atoms on benzene ring.

The Radial distribution function (RDF) refers to the probability of distribution of other particles in the space around a given particle, that is, the distance from other particles to a given particle. RDF can be used to study the ordering of materials from the structural characteristics of micro-reactive materials [30]. The definition is as follows:

(9) $$g_{AB}\left(r\right)=\frac{V\times \sum_{i\neq j}\delta \left(r-\left|r_{Ai}-r_{Bi}\right|\right)}{\left(N_{A}N_{B}-N_{AB}\right)4\pi r^{2}\mathrm{dr}}$$

where *N_A_* and *N_B_* are the number of atoms in groups *A* and *B* respectively, *N_AB_* is the number of atoms shared by the two groups of atoms, r is the radial distance coordinate, and atoms *i* and *j* are the atoms in groups *A* and *B* respectively. Figure 8 shows the RDF of PMIA systems at different temperatures, and Figure 9 shows the local structure during the temperature change process.

In Figure 8a, by comparing the RDF curves of the model at different temperatures, there is no obvious difference in the RDF of the system at different temperatures, showing similar characteristics. Due to the van der Waals volume exclusion effect between atoms, the value of RDF is 0 when R is less than 0.9 Å. Due to the existence of N-H key in the model, the first peak appears at 1.01 Å. However, with the increase of temperature, the bond length between hydroxyl groups gradually increases and converges with the subsequent peak value, so that the peak value at 1.01 Å gradually decreases. The covalent bonds between many hydrogen atoms and carbon atoms in the model make a peak appear at 1.10 Å. With the increase of temperature, the x value corresponding to the peak increases and the peak decreases. This is because the increase in bond length caused by temperature makes the peak shift to the right. The two peaks at about 1.21 Å and 1.39 Å correspond to the covalent bond between the carbon atom (c) and the non-hydrogen atom (as shown in Figure 9c,d); The subsequent peaks correspond to atoms that are not directly connected, such as the distance between the terminal carbon and carbon of the C-C-C series of benzene ring, as shown in Figure 9d (about 2.45 Å).

In Figure 8b, the RDFs of pure PMIA and SiO_2_/PMIA composite models are calculated. It can be seen from the figure that the general trend of all atom RDFs of the two models is the same and basically coincides. Because the proportion of oxygen atoms (O) and silicon atoms (Si) in the nano filler is small, it does not make a significant contribution to the corresponding peak, and there is only one peak at 3.2 Å.

It is worth noting that G(R) does not have any peak in the range greater than 3.8 Å and tends to be close to 1, which is generally considered as evidence that the polymer system has amorphous characteristics [31]. In general, the doping of nano SiO_2_ has no significant effect on the total atoms RDF of the system.

## 5. Conclusions

Focusing on the aramid insulating materials used in power systems equipment, this paper studies the influence of temperature on the thermodynamic properties of PMIA and SiO_2_-modified PMIA materials within the operating temperature range of the equipment, and obtains the following conclusions:

The addition of nano-SiO_2_ in the PMIA can increase the thermal conductivity, glass transition temperature, Young’s modulus, and Shear modulus, and decrease the thermal expansion coefficient of the PMIA. It can be seen that the addition of SiO_2_ can improve the thermal conductivity, thermal properties, and mechanical properties of the PMIA.

The Young’s modulus and Shear modulus of pure PMIA decreased by 16.8% at 450 K, compared with that at 250 K, while the Young’s modulus and Shear modulus of SiO_2_/PMIA composites decreased by 20.9% and 22.2%, respectively. The mechanical properties of modified aramid composites deteriorated with the increase of temperature, but the mechanical properties of modified aramid composites were always better than those of pure PMIA composites in the range of the temperature rise of the casing.

The thermal stability of aramid materials in the glass state is better than that in the rubber state. After the addition of SiO_2_, the CTE of PMIA will decrease in both the glass and rubber states, and the thermal stability of the composite will be improved. This is because the addition of SiO_2_ will hinder the movement of the aramid molecular chain.

The addition of SiO_2_ hinders the movement of the molecular chain and changes the microstructure of the PMIA, which is reflected in the fact that the free volume distribution of the PMIA is less easily dispersed with the increase of temperature.

## Figures and Tables

**Table 1:** Thermal conductivity versus the nano-SiO_2_ radius.

Radius of Nano-SiO_2_	0 Å	4 Å	8 Å	12 Å
Thermal conductivity/(W m^−1^ K^−1^)	0.249	0.286	0.396	0.387

**Table 2:** Free volume ratio of the PMIA and SiO_2_/PMIA models at different temperatures.

	300 K	350 K	400 K	450 K	500 K
*V_o_/*Å^3^	39,409.69	39,756.95	39,717.33	39,661.39	39,599.70
*V_f_/*Å^3^	6356.44	6517.82	6781.41	7738.20	8201.36
*FFV_PMIA_*	0.139	0.141	0.146	0.163	0.172
*V_o_/*Å^3^	41,472.52	41,421.98	41,609.79	41,510.58	41,735.27
*V_f_/*Å^3^	5537.94	6134.32	5977.88	6386.57	6318.73
*FFV* *_SiO2_*	0.118	0.123	0.126	0.133	0.135

## Data Availability

The data presented in this study are available in Molecular Dynamics Simulation of Thermodynamic Properties of Nano Silica/PMIA Composites.
